# In memoriam: Leendert P. van Ofwegen (1953–2021), octocoral taxonomist

**DOI:** 10.3897/zookeys.1052.71435

**Published:** 2021-07-30

**Authors:** Bert W. Hoeksema

**Affiliations:** 1 Naturalis Biodiversity Center, PO Box 9517, 2300 RA Leiden, The Netherlands Naturalis Biodiversity Center Leiden Netherlands; 2 Groningen Institute for Evolutionary Life Sciences, University of Groningen, P.O. Box 11103, 9700 Groningen, The Netherlands University of Groningen Groningen Netherlands

## Abstract

none

Leendert Pieter van Ofwegen passed away on 25 June 2021 at the age of 68 years. He had been diagnosed with lung cancer in late 2020, which in June this year appeared to be in complete remission. Tragically, there was a relapse. His health suddenly worsened while he was on holiday in Marbella (Spain) and the disease rapidly led to his death. With his passing, the scientific community loses a great and devoted octocoral taxonomist who was a good friend to his colleagues and a dedicated mentor to his students.

Leendert (“Leen”) was born in Leiden on 22 April 1953. From 1959 to 1965 he attended primary school (“Rozenlaanschool”) in Boskoop, a town in the province of South Holland, where he lived with his parents and elder brother while he grew up. Boskoop is famous for its woody plant and perennial nurseries, which employed many local people, including Leen’s father. Leen attended secondary school in Alphen aan den Rijn (“Christelijk Lyceum”) from 1965 to 1970, after which he studied biology at Leiden University from 1970 until 1980. His study was interrupted by military service, which was compulsory in the Netherlands at that time.

Leen obtained a BSc in 1975 with geology as second major and continued on to do an MSc, of which two projects stand out. Leen’s first research project concerned Devonian brachiopods, for which he excavated fossils in the Cantabrian mountains, northwestern Spain. This research took more time than anticipated but eventually became part of Leen’s first co-authored publication (Krans et al. 1982). Apparently, he did not consider this work relevant to his career because he always left it out of his CV (e.g., van Ofwegen 2007). For the second research project, Leen became an intern at the Rijksmuseum van Natuurlijke Historie (RMNH, presently Naturalis Biodiversity Center), which at that time was a centre of systematic research on Cnidaria. He worked under supervision of Koos den Hartog (curator of “Coelenterata”), who proposed that Leen could work on the taxonomy of octocorals from the Saba Bank, which were collected during an expedition to the former Netherlands Antilles, currently the Dutch Caribbean (van der Land 1972; Hoeksema et al. 2017). The work was not published but the specimens were identified and are present in the RMNH.Coel collection in Leiden, where they are available for future research (Hoeksema et al. 2011). Leen’s internship at the RMNH was the start of his interest and expertise in octocoral taxonomy.

Following his MSc, as no jobs were available in octocoral taxonomy, Leen did free-lance work as a computer programmer but continued his research in his spare time, under the supervision of Koos den Hartog, while he also exchanged ideas with Hydrozoa specialist Prof. Willem Vervoort (Figure 2e). During this period, Leen started to work on a collection of gorgonians collected by the International Indian Ocean Expedition of *R/V Anton Bruun* (1963 and 1964). That collection belonged to the Invertebrate Zoology department of the Smithsonian Institution in Washington, D.C. (USNM), and was on loan to the RMNH to make it available for research by octocoral expert Dr. Jacob Verseveldt. Verseveldt, however, was mostly interested in studies of fleshy octocorals (den Hartog 1987) rather than gorgonians, which may explain why this collection remained untouched for several years. Someone had to do the job and Leen made himself available. After the study (van Ofwegen 1990), the collection was returned to Washington.

Following the suggestion of Koos den Hartog, Leen decided to follow Jacob Verseveldt in his work on the taxonomy of soft corals, for which the same methodology was used as in the taxonomy of gorgonians. This methodology consisted of morphological examination of the coral colonies and in particular analyses of their sclerites, being microscopic calcite skeletal elements embodied in the soft tissue of the animals. At first, Leen made hand drawings with a light microscope and a *camera lucida* but later he made extensive use of high-resolution scanning electronic microscopy (SEM). At a later stage, he also became involved in molecular analyses applied to the taxonomy of octocorals, as shown by co-authored publications with colleagues in Leiden and from abroad (e.g., McFadden et al. 2006; van Ofwegen and Groenenberg 2007; McFadden and van Ofwegen 2013; Reijnen et al. 2014).

In 1992, Leen got a position as manager of the editorial office of the RMNH museum journals, Zoologische Mededelingen and Zoologische Verhandelingen, and continued publishing papers on octocorals in his spare time. Owing to Leen’s growing publication record, his reputation as world-renowned octocoral taxonomist began to attract attention and he was approached by several young scientists who wanted to be trained in his field of research. Leen’s first student was Dr. Jayasree Vennam from the National Institute of Oceanography (NIO) at Dona Paula, Goa, India (Figure 2e), who stayed six months in Leiden as a visiting scientist during 1989–1990 (den Hartog and Vennam 1993). One reason for this interest in octocorals was their major role as producers of novel chemicals for the design of medicinal compounds. This pharmacological application of octocoral taxonomy resulted in Leen’s co-authorships of various publications on marine natural products derived from species that he undertook to identify (Appendix 2). In these projects, he mainly collaborated with Dr. R. Andersen (Canada), Dr. M. Jaspars (United Kingdom), Dr. Wenhan Lin (China), and Prof. Dr. P. Proksch (Germany).

In 2001, Leen became employed as curator of coelenterates at the Leiden museum, succeeding Koos den Hartog, who passed away in 2000 (van der Land 2003). Leen’s main research was focused on (1) a taxonomic revision of the soft-coral genera *Litophyton*, *Nephthea*, and *Stereonephthya* (family Nephtheidae), which resulted in the establishment of *Chromonephthea* and many new species in that genus (Figure 5c; van Ofwegen 2005), and (2) on collections made in the Indo-West Pacific centre of maximum octocoral biodiversity. For this research he made use of the octocoral collection in the Leiden museum, but also those in Berlin and Washington, DC, which he visited in the company of stony-coral specialist and co-worker Bert Hoeksema. Various colleagues from abroad regularly came to Leiden to work with Leen on the octocoral collections, such as Dr. Yehuda Benayahu (Israel), Dr. Tatiana Dautova (Russia), Dr. Asako K. Matsumoto (Japan; Figure 2f), and Dr. Cathy McFadden (USA). These visits resulted in several co-authored publications (Appendix 2), covering a large variety of biogeographic regions. Undoubtedly, Leen has become a world authority on octocoral taxonomy and was approached by numerous colleagues for his expertise. His name is mentioned in many field guides, acknowledging his help in the identification of species illustrated in photographs.

Octocoral material in museum collections is either stored in ethanol or dried, both of which cause specimens to lose their original shape and often their colour. In order to learn about the natural habitus of soft corals, Leen decided that he had to learn diving with the use of SCUBA. This enabled him to participate in fieldwork sampling during surveys and large-scale expeditions in the company of his colleagues of the Naturalis marine research team (“Naturalis Zeeteam”), even before he got a position as a curator (Figures 1, 3, 4): in the Seychelles (December 1992–January 1993), South Sulawesi (1994), North Sulawesi (1994), Ambon (1996), South Sulawesi (1998), Cebu, Philippines (1999), Bali (2001), East Kalimantan (2003), Thousand Islands (2005), Palau (2005), and Raja Ampat (2007).

**Figure 1.**
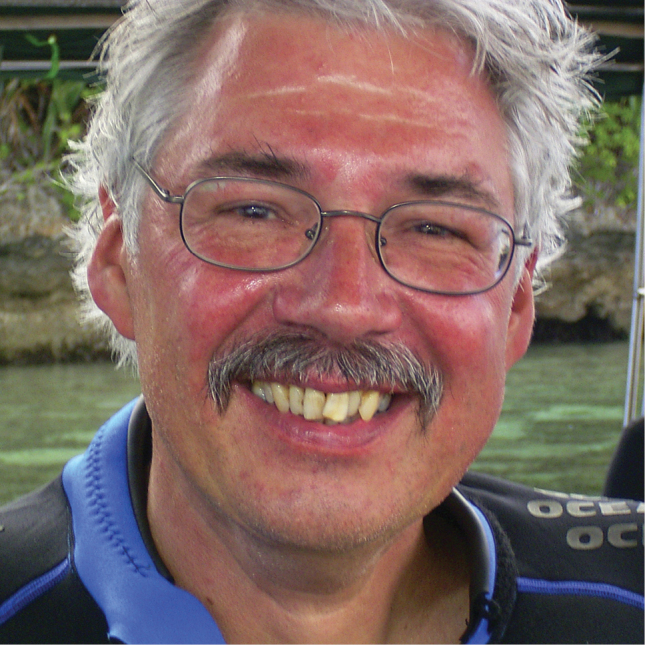
Portrait of Leen van Ofwegen during fieldwork in Indonesia (2003). Photograph credit: author.

Most of these expeditions were in Indonesia and in this way Leen established a close collaboration with two octocoral scientists from the Research Centre for Oceanography, Indonesian Institute of Sciences, Jakarta: M.I.Y.T. Hermanlimianto (Yosephine Tuti), specialised in gorgonians, and Anna E.W. Manuputty, specialised in soft corals. Both of them also visited Leen and his collection; Yosephine did so several times (Figure 2c). All in all, Leen spent much time on collection work for which he collaborated with collection managers Chiel Slierings (who retired in October 2007) and Koos van Egmond until Leen’s own retirement on 31 December 2016.

**Figure 2.**
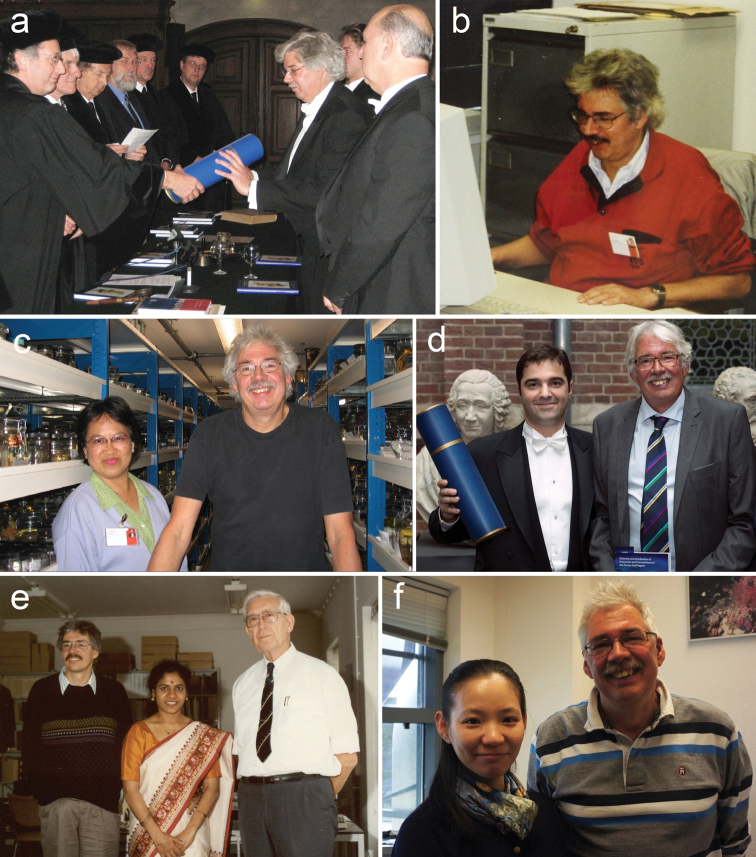
Photographs of Leen in Leiden **a** receiving his PhD degree (2007) **b** in his office (1998) **c** in the wet “Coelenterata” collection with Yosphine Tuti from Indonesia (2013) **d** as co-supervisor of Kaveh Samimi-Namin (2016)**e** with Jayasree Vennam from India and Prof. Willem Vervoort (1990) **f** in his office with Asako Matsumoto from Japan (2012). Photograph credits **a** Jacques van Ofwegen **b** unknown **c** author **d** Livia Oliveira **e** unknown **f** Bastian Reijnen.

In order to strengthen his international contacts, Leen attended several international conferences and workshops to meet with other octocoral workers (Figure 5), such as the 6^th^ International Conference on Coelenterate Biology, Noordwijkerhout, The Netherlands (1996); the 9^th^ International Coral Reef Symposium Bali, Indonesia (2000); the 2^nd^ International Workshop on Octocoral Taxonomy, Darwin, Australia (2002); the 7^th^ International Conference on Coelenterate Biology, Lawrence, Kansas, USA (2003); the 10^th^ International Coral Reef Symposium, Okinawa, Japan (2004); the 1^st^ Asia Pacific Coral Reef Symposium, Hong Kong (2006); the 2^nd^ Asia Pacific Coral Reef Symposium, Phuket Thailand (2010); and the 8^th^ International Conference on Coelenterate Biology, Eilat, Israel (2013). He acted as an instructor with other colleagues during two octocoral taxonomy workshops: in Trivandrum, Kerala, India (2005) and in Phuket, Thailand (2007).

Leen’s study of the octocoral family Nephtheididae was also the subject of his PhD from Leiden University, obtained on 6 November 2007 (Figure 2a), under the supervision of Prof. Dr. Edi Gittenberger and Dr. B.W. Hoeksema (van Ofwegen 2007). He coached and supervised two PhD candidates himself and became co-supervisor in 2016 (together with Bert Hoeksema) for Kaveh Samimi-Namin (Samimi-Namin 2016; Figure 2d) and Bastian T. Reijnen (Reijnen 2016), both at Leiden University under supervision of Edi Gittenberger. Leen also supervised two MSc students from Leiden University, Frank R. Stokvis and Yee Wah Lau.

In 2001, Leen also succeeded Koos den Hartog in his capacity of editor-in-chief of the two zoological museum journals until these ceased publication. In 2004, Zoologische Verhandelingen became incorporated in Zoologische Mededelingen, which stopped publication in 2014. In 2008, Leen became an associate editor for ZooKeys (with a focus on octocorals and other anthozoans). In the same year he also became editor of Octocorallia for the World Register of Marine Species, now also accessible through the World List of Octocorallia (Cordeiro et al. 2021), with which he first collaborated with Dr. Gary Williams, who oversaw the sea pen data (Pennatulacea). After his retirement, Leen stopped his editorial tasks, but he continued publishing papers until his death. His dedication and expertise in octocoral taxonomy is demonstrated by an impressive series of publications and a large collection of specimens. In honour of Leen’s octocoral expertise one species was named after him, *Fasciclia ofwegeni* Janes, 2008, based on type material collected by Leen in the Seychelles (Janes 2008).

**Figure 3.**
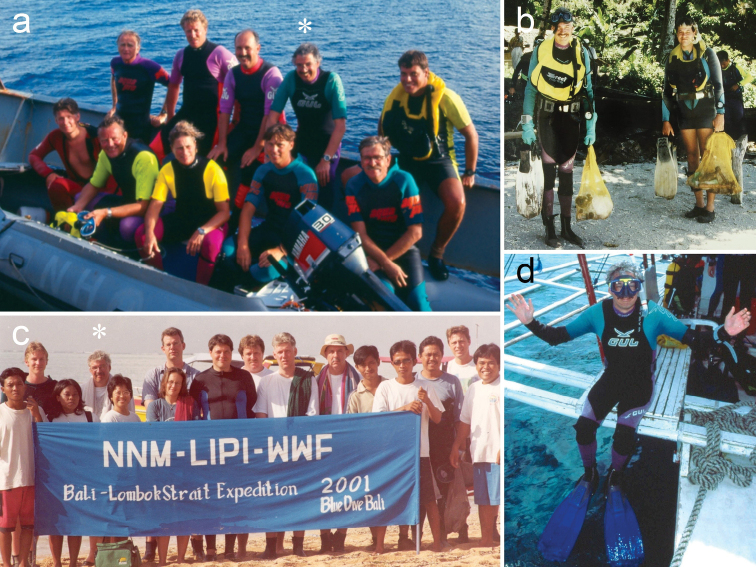
Photographs of Leen (*) during fieldwork **a** with colleagues on board *R/V Tyro* in the Seychelles (1993) **b** with Bert Hoeksema in Ambon, Indonesia (1996) **c** with colleagues in Bali, Indonesia (2001) **d** in Cebu, the Philippines (1999). Photograph credits **a** Willem Kolvoort **b** unknown **c** unknown **d** author.

Leen described many new taxa himself or with co-authors, including seven families, 25 genera, and 214 species, ranging in publication years from 1987 until the present (Appendix 1). Various new species were named after Naturalis colleagues (*Chromonephthea egmondi*, *C. franseni*, *C. goudi*, *C. hoeksemai*, *C. slieringsi*, *Dendronephthya vervoorti*, *Lignella hartogi*, *Sinularia vanderlandi*), and several others were named after octocoral specialists (*Chromonephthea aldersladei*, *C. bayeri*, *C. benayahui*, *C. cairnsi*, *C. grasshoffi*, *C. tentoriae*, *C. williamsi*, *Sinularia verseveldti*, *Wrightella stiasnyi*). Having known Leen for more than 35 years, it is beyond doubt that he will be missed by his colleagues and students, who will remember him not only for his life-time dedication to octocoral taxonomy and his vast knowledge on that topic, but also for his characteristic smile (Figures 1–5), unique sense of humour, and heartfelt friendship.

**Figure 4.**
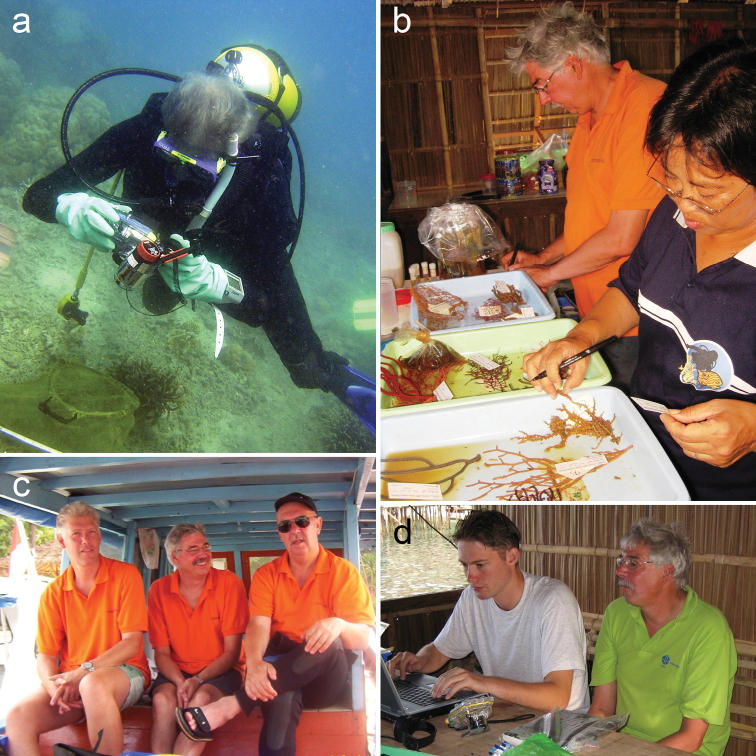
Photographs of Leen during fieldwork in Indonesia (continued) **a** photography and sampling underwater off Jakarta in the Java Sea (2005) **b** preparing specimens with Yosephine Tuti in Raja Ampat (2007) **c** with colleagues Charles Fransen and Chiel Slierings on board of a small wooden boat during a survey in the Thousand Islands, Indonesia (2005) **d** Processing data with Frank Stokvis on Kri Island, Raja Ampat (2007). Photograph credits **a** author **b** Stefano Draisma **c** Koos van Egmond **d** unknown.

**Figure 5.**
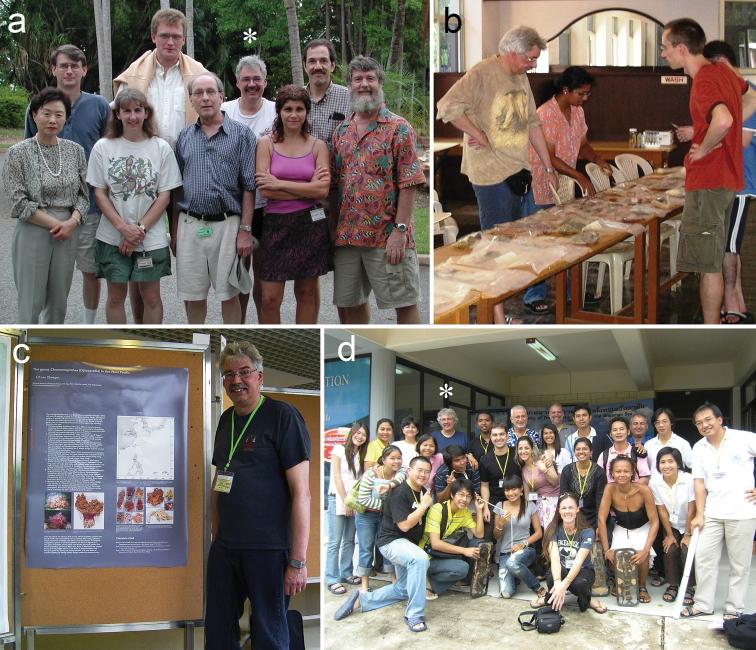
Photographs of Leen (*) during conferences and workshops **a** with other participants of the 2^nd^ International Workshop on Octocoral Taxonomy, Darwin, Australia (2002): Stephen Cairns, Götz Reinicke, Gary Williams, Jun-Im Song, Catherine McFadden, Manfred Grasshoff, Philip Alderslade **b** discussing octocoral samples with Anita George, Jaret Bilewitch, and Kaveh Samimi-Namin during the International Workshop on the Taxonomy of Octocorals in Trivandrum, Kerala, India (2005) **c** presenting a poster during the 1^st^ Asia Pacific Coral reef Symposium, Hong Kong (2006) **d** with other participants during the 4^th^ octocoral taxonomy workshop in Phuket, Thailand (2007). Photograph credits **a** museum and Art Galleries of the Northern Territories in Darwin **b** unknown **c** author **d** unknown.

## Appendix 1

Octocoral taxa described by Leen P. van Ofwegen (and co-authors). Source: Cordeiro et al. (2021)

**Number of families: 7**

**2010**

Acanthoaxiidae van Ofwegen &amp; McFadden, 2010

**2012**

Aquaumbridae Breedy, van Ofwegen &amp; Vargas, 2012

Arulidae McFadden &amp; van Ofwegen, 2012

**2013**

Parasphaerascleridae McFadden &amp; van Ofwegen, 2013

**2017**

Acrophytidae McFadden &amp; van Ofwegen, 2017

Corymbophytidae McFadden &amp; van Ofwegen, 2017

Leptophytidae McFadden &amp; van Ofwegen, 2017

**Number of genera: 25**

**1991**

*Pseudothelogorgia* van Ofwegen, 1991

**1997**

*Leptophyton* van Ofwegen &amp; Schleyer, 1997

**2003**

*Canarya* Ocaña &amp; van Ofwegen, 2003

*Denhartogia* Ocaña &amp; van Ofwegen, 2003

**2005**

*Chromonephthea* van Ofwegen, 2005

**2006**

*Ceeceenus* van Ofwegen &amp; Benayahu, 2006

**2007**

*Incrustatus* van Ofwegen, Häussermann &amp; Försterra, 2007

**2010**

*Acanthoaxis* van Ofwegen &amp; McFadden, 2010

**2011**

*Stragulum* van Ofwegen &amp; Haddad, 2011

**2012**

*Aquaumbra* Breedy, van Ofwegen &amp; Vargas, 2012

*Arula* McFadden &amp; van Ofwegen, 2012

*Inconstantia* McFadden &amp; van Ofwegen, 2012

**2013**

*Parasphaerasclera* McFadden &amp; van Ofwegen, 2013

*Sphaerasclera* McFadden &amp; van Ofwegen, 2013

**2014**

*Complexum* van Ofwegen, Aurelle &amp; Sartoretto, 2014

**2017**

*Circularius* McFadden &amp; van Ofwegen, 2017

*Corymbophyton* McFadden &amp; van Ofwegen, 2017

*Porphyrophyton* McFadden &amp; van Ofwegen, 2017

*Tenerodus* McFadden &amp; van Ofwegen, 2017

**2018**

*Caementabunda* Benayahu, van Ofwegen &amp; McFadden, 2018

*Conglomeratusclera* Benayahu, van Ofwegen &amp; McFadden, 2018

*Hana* Lau, Stokvis, van Ofwegen &amp; Reimer, 2018 [accepted as *Hanah* Lau, Stokvis, van Ofwegen &amp; Reimer, 2020]

**2020**

*Hanah* Lau, Stokvis, van Ofwegen &amp; Reimer, 2020

**2021**

*Rhodolitica* Breedy, van Ofwegen, McFadden &amp; Murillo-Cruz, 2021

*Unomia* Benayahu, van Ofwegen, Allais &amp; McFadden, 2021

**Number of species: 214**

**1987**

*Acabaria andamanensis* van Ofwegen, 1987 [accepted as *Melithaea andamanensis* (van Ofwegen, 1987)]

*Clathraria maldivensis* van Ofwegen, 1987 [accepted as *Melithaea maldivensis* (van Ofwegen, 1987)]

*Clathraria omanensis* van Ofwegen, 1987 [accepted as *Melithaea omanensis* (van Ofwegen, 1987)]

**1989**

*Wrightella stiasnyi* van Ofwegen, 1989 [accepted as *Melithaea stiasnyi* (van Ofwegen, 1989)]

**1990**

*Lignella hartogi* van Ofwegen, 1990 [accepted as *Pseudothelogorgia hartogi* (van Ofwegen, 1990)]

**1991**

*Dendronephthya bruuni* Verseveldt &amp; van Ofwegen, 1991

*Dendronephthya hystricosa* Verseveldt &amp; van Ofwegen, 1991

*Dendronephthya pyriformis* Verseveldt &amp; van Ofwegen, 1991

*Dendronephthya staphyloidea* Verseveldt &amp; van Ofwegen, 1991

*Dendronephthya vervoorti* Verseveldt &amp; van Ofwegen, 1991

*Sinularia abhishiktae* van Ofwegen &amp; Vennam, 1991

**1992**

*Alcyonium compactofestucum* Verseveldt &amp; van Ofwegen, 1992

*Alcyonium rudyi* Verseveldt &amp; van Ofwegen, 1992 [accepted as *Discophyton rudyi* (Verseveldt &amp; van Ofwegen, 1992)]

*Alcyonium senegalense* Verseveldt &amp; van Ofwegen, 1992

*Alcyonium spitzbergense* Verseveldt &amp; van Ofwegen, 1992

*Cladiella daphnae* van Ofwegen &amp; Benayahu, 1992

*Sinularia platylobata* van Ofwegen &amp; Benayahu, 1992

**1994**

*Sinularia slieringsi* van Ofwegen &amp; Vennam, 1994

**1996**

*Sinularia verseveldti* van Ofwegen, 1996

**1997**

*Alcyonium grandis* Casas, Ramil &amp; van Ofwegen, 1997

*Alcyonium paucilobulatum* Casas, Ramil &amp; van Ofwegen, 1997

*Alcyonium southgeorgiensis* Casas, Ramil &amp; van Ofwegen, 1997

*Leptophyton benayahui* van Ofwegen &amp; Schleyer, 1997

**1999**

*Lobophytum jasparsi* van Ofwegen, 1999

**2001**

*Astrogorgia bayeri* van Ofwegen &amp; Hoeksema, 2001

*Sinularia vanderlandi* van Ofwegen, 2001

**2003**

*Denhartogia hartogi* Ocaña &amp; van Ofwegen, 2003

**2005**

*Chromonephthea aldersladei* van Ofwegen, 2005

*Chromonephthea bayeri* van Ofwegen, 2005

*Chromonephthea benayahui* van Ofwegen, 2005

*Chromonephthea braziliensis* van Ofwegen, 2005

*Chromonephthea brevis* van Ofwegen, 2005

*Chromonephthea cairnsi* van Ofwegen, 2005

*Chromonephthea cobourgensis* van Ofwegen, 2005

*Chromonephthea egmondi* van Ofwegen, 2005

*Chromonephthea exosis* van Ofwegen, 2005

*Chromonephthea franseni* van Ofwegen, 2005

*Chromonephthea frondosa* van Ofwegen, 2005

*Chromonephthea fruticosa* van Ofwegen, 2005

*Chromonephthea goudi* van Ofwegen, 2005

*Chromonephthea grandis* van Ofwegen, 2005

*Chromonephthea grasshoffi* van Ofwegen, 2005

*Chromonephthea hoeksemai* van Ofwegen, 2005

*Chromonephthea hornerae* van Ofwegen, 2005

*Chromonephthea imaharai* van Ofwegen, 2005

*Chromonephthea imperfecta* van Ofwegen, 2005

*Chromonephthea levis* van Ofwegen, 2005

*Chromonephthea megasclera* van Ofwegen, 2005

*Chromonephthea minor* van Ofwegen, 2005

*Chromonephthea muironensis* van Ofwegen, 2005

*Chromonephthea obscura* van Ofwegen, 2005

*Chromonephthea ostrina* van Ofwegen, 2005

*Chromonephthea palauensis* van Ofwegen, 2005

*Chromonephthea rotunda* van Ofwegen, 2005

*Chromonephthea simulata* van Ofwegen, 2005

*Chromonephthea singularis* van Ofwegen, 2005

*Chromonephthea slieringsi* van Ofwegen, 2005

*Chromonephthea spinosa* van Ofwegen, 2005

*Chromonephthea tentoriae* van Ofwegen, 2005

*Chromonephthea variabilis* van Ofwegen, 2005

*Chromonephthea williamsi* van Ofwegen, 2005

**2006**

*Alcyonium megasclerum* Stokvis &amp; van Ofwegen, 2006

*Alcyonium profundum* Stokvis &amp; van Ofwegen, 2006

*Alcyonium rubrum* Stokvis &amp; van Ofwegen, 2006 [accepted as *Alcyonium burmedju* Sampaio, Stokvis &amp; van Ofwegen, 2016]

*Astrogorgia balinensis* Hermanlimianto &amp; van Ofwegen, 2006

*Ceeceenus levis* van Ofwegen &amp; Benayahu, 2006

*Ceeceenus pannosus* van Ofwegen &amp; Benayahu, 2006

*Ceeceenus quadrus* van Ofwegen &amp; Benayahu, 2006

*Ceeceenus torus* van Ofwegen &amp; Benayahu, 2006

**2007**

*Alcyonium glaciophilum* van Ofwegen, Häussermann &amp; Försterra, 2007

*Alcyonium jorgei* van Ofwegen, Häussermann &amp; Försterra, 2007

*Alcyonium roseum* van Ofwegen, Häussermann &amp; Försterra, 2007 [accepted as *Alcyonium varum* McFadden &amp; van Ofwegen, 2013]

*Alcyonium yepayek* van Ofwegen, Häussermann &amp; Försterra, 2007

*Alertigorgia hoeksemai* van Ofwegen &amp; Alderslade, 2007

*Incrustatus comauensis* van Ofwegen, Häussermann &amp; Försterra, 2007

*Sinularia acuta* Manuputty &amp; van Ofwegen, 2007

*Sinularia corpulentissima* Manuputty &amp; van Ofwegen, 2007

*Sinularia curvata* Manuputty &amp; van Ofwegen, 2007

*Sinularia kotanianensis* Manuputty &amp; van Ofwegen, 2007

*Sinularia longula* Manuputty &amp; van Ofwegen, 2007

**2008**

*Sinularia babeldaobensis* van Ofwegen, 2008

*Sinularia bisulca* van Ofwegen, 2008

*Sinularia bremerensis* van Ofwegen, 2008

*Sinularia confusa* van Ofwegen, 2008

*Sinularia crebra* van Ofwegen, 2008

*Sinularia diffusa* van Ofwegen, 2008

*Sinularia digitata* van Ofwegen, 2008

*Sinularia finitima* van Ofwegen, 2008

*Sinularia flaccida* van Ofwegen, 2008

*Sinularia foliata* van Ofwegen, 2008

*Sinularia humilis* van Ofwegen, 2008

*Sinularia linnei* van Ofwegen, 2008

*Sinularia luxuriosa* van Ofwegen, 2008

*Sinularia papula* van Ofwegen, 2008

*Sinularia siaesensis* van Ofwegen, 2008

*Sinularia sublimis* van Ofwegen, 2008

*Sinularia tumulosa* van Ofwegen, 2008

*Sinularia ultima* van Ofwegen, 2008

*Sinularia uniformis* van Ofwegen, 2008

*Sinularia verruca* van Ofwegen, 2008

*Sinularia woodyensis* van Ofwegen, 2008

**2009**

*Astrogorgia fruticosa* Samimi Namin &amp; van Ofwegen, 2009

*Euplexaura plana* Samimi Namin &amp; van Ofwegen, 2009

*Lobophytum mortoni* Benayahu &amp; van Ofwegen, 2009

*Sarcophyton minusculum* Samimi Namin &amp; van Ofwegen, 2009

*Sarcophyton tumulosum* Benayahu &amp; van Ofwegen, 2009

**2010**

*Acanthoaxis wirtzi* van Ofwegen &amp; McFadden, 2010

*Bebryce inermis* Samimi Namin &amp; van Ofwegen, 2010

*Sinularia capricornis* Dautova, van Ofwegen &amp; Savinkin, 2010

*Sinularia multiflora* Dautova, van Ofwegen &amp; Savinkin, 2010

*Sinularia pumila* Dautova, van Ofwegen &amp; Savinkin, 2010

*Sinularia sarmentosa* Dautova, van Ofwegen &amp; Savinkin, 2010

*Sinularia torta* Dautova, van Ofwegen &amp; Savinkin, 2010

*Sinularia uva* Dautova, van Ofwegen &amp; Savinkin, 2010

**2011**

*Lobophytum hsiehi* Benayahu &amp; van Ofwegen, 2011

*Sinularia choui* Benayahu &amp; van Ofwegen, 2011

*Sinularia daii* Benayahu &amp; van Ofwegen, 2011

*Sinularia soongi* Benayahu &amp; van Ofwegen, 2011

*Stragulum bicolor* van Ofwegen &amp; Haddad, 2011

**2012**

*Aquaumbra klapferi* Breedy, van Ofwegen &amp; Vargas, 2012

*Arula petunia* McFadden &amp; van Ofwegen, 2012

*Cornularia pabloi* McFadden &amp; van Ofwegen, 2012

*Eunephthya celata* McFadden &amp; van Ofwegen, 2012

*Eunephthya ericius* McFadden &amp; van Ofwegen, 2012

*Eunephthya granulata* McFadden &amp; van Ofwegen, 2012

*Eunephthya shirleyae* McFadden &amp; van Ofwegen, 2012

*Inconstantia exigua* McFadden &amp; van Ofwegen, 2012

*Inconstantia pannucea* McFadden &amp; van Ofwegen, 2012

*Inconstantia procera* McFadden &amp; van Ofwegen, 2012

*Sinularia penghuensis* van Ofwegen &amp; Benayahu, 2012

*Sinularia shlagmani* Benayahu &amp; van Ofwegen, 2012

*Sinularia tessieri* Benayahu &amp; van Ofwegen, 2012

*Sinularia wanannensis* van Ofwegen &amp; Benayahu, 2012

**2013**

*Alcyonium varum* McFadden &amp; van Ofwegen, 2013

*Incrustatus niarchosi* McFadden &amp; van Ofwegen, 2013

*Sinularia australiensis* van Ofwegen, Benayahu &amp; McFadden, 2013

*Sinularia eilatensis* van Ofwegen, Benayahu &amp; McFadden, 2013

**2014**

*Complexum pusillum* van Ofwegen, Aurelle &amp; Sartoretto, 2014

*Melithaea hendersoni* Reijnen, McFadden, Hermanlimianto &amp; van Ofwegen, 2014

*Melithaea mcqueeni* Reijnen, McFadden, Hermanlimianto &amp; van Ofwegen, 2014

*Melithaea shanni* Reijnen, McFadden, Hermanlimianto &amp; van Ofwegen, 2014

*Melithaea thorpeae* Reijnen, McFadden, Hermanlimianto &amp; van Ofwegen, 2014

*Melithaea wrighti* Reijnen, McFadden, Hermanlimianto &amp; van Ofwegen, 2014

**2015**

*Melithaea boninensis* Matsumoto &amp; van Ofwegen, 2015

*Melithaea doederleini* Matsumoto &amp; van Ofwegen, 2015

*Melithaea isonoi* Matsumoto &amp; van Ofwegen, 2015

*Melithaea keramaensis* Matsumoto &amp; van Ofwegen, 2015

*Melithaea oyeni* Matsumoto &amp; van Ofwegen, 2015

*Melithaea ryukyuensis* Matsumoto &amp; van Ofwegen, 2015

*Melithaea sagamiensis* Matsumoto &amp; van Ofwegen, 2015

*Melithaea satsumaensis* Matsumoto &amp; van Ofwegen, 2015

*Melithaea suensoni* Matsumoto &amp; van Ofwegen, 2015

*Melithaea tanseii* Matsumoto &amp; van Ofwegen, 2015

*Melithaea tokaraensis* Matsumoto &amp; van Ofwegen, 2015

**2016**

*Alcyonium burmedju* Sampaio, Stokvis &amp; van Ofwegen, 2016

*Bebryce asteria* Bayer &amp; van Ofwegen, 2016

*Bebryce cofferi* Bayer &amp; van Ofwegen, 2016

*Bebryce otsuchiensis* Matsumoto &amp; van Ofwegen, 2016

*Bebryce rotunda* Matsumoto &amp; van Ofwegen, 2016

*Bebryce satsumaensis* Matsumoto &amp; van Ofwegen, 2016

*Briareum cylindrum* Samimi-Namin &amp; van Ofwegen, 2016

*Euplexaura yayoii* Matsumoto &amp; van Ofwegen, 2016

*Litophyton curvum* van Ofwegen, 2016

*Melithaea davidi* Samimi-Namin, van Ofwegen &amp; McFadden, 2016

*Sinularia levi* van Ofwegen, McFadden &amp; Benayahu, 2016

*Trimuricea bicolor* Samimi-Namin &amp; van Ofwegen, 2016

*Trimuricea flava* Samimi-Namin &amp; van Ofwegen, 2016

*Trimuricea omanensis* Samimi-Namin &amp; van Ofwegen, 2016

*Trimuricea persica* Samimi-Namin &amp; van Ofwegen, 2016

*Trimuricea spinosa* Samimi-Namin &amp; van Ofwegen, 2016

*Trimuricea tuberculosa* Samimi-Namin &amp; van Ofwegen, 2016

**2017**

*Alcyonium dolium* McFadden &amp; van Ofwegen, 2017

*Lampophyton spinatum* McFadden &amp; van Ofwegen, 2017

*Leptophyton fustis* McFadden &amp; van Ofwegen, 2017

*Sinularia mesophotica* Benayahu, McFadden, Shoham &amp; van Ofwegen, 2017

*Tenerodus pollex* McFadden &amp; van Ofwegen, 2017

**2018**

*Dendronephthya perezi* Cordeiro &amp; van Ofwegen, 2018

*Hana hanagasa* Lau, Stokvis, van Ofwegen &amp; Reimer, 2018 [accepted as *Hanah hanagasa* (Lau, Stokvis, Ofwegen &amp; Reimer, 2018)]

*Hana hanataba* Lau, Stokvis, van Ofwegen &amp; Reimer, 2018 [accepted as *Hanah hanataba* (Lau, Stokvis, Ofwegen &amp; Reimer, 2018)]

**2019**

*Calcigorgia gigantea* Matsumoto, van Ofwegen &amp; Bayer, 2019

*Calcigorgia gracilis* Matsumoto, van Ofwegen &amp; Bayer, 2019

*Calcigorgia pacifica* Matsumoto, van Ofwegen &amp; Bayer, 2019

**2020**

*Litophyton acutum* van Ofwegen, 2020

*Litophyton brachiatum* van Ofwegen, 2020

*Litophyton brewerensis* van Ofwegen, 2020

*Litophyton burfordensis* van Ofwegen, 2020

*Litophyton carnatum* van Ofwegen, 2020

*Litophyton cockburnensis* van Ofwegen, 2020

*Litophyton darleyensis* van Ofwegen, 2020

*Litophyton daviesensis* van Ofwegen, 2020

*Litophyton dipensis* van Ofwegen, 2020

*Litophyton elfordensis* van Ofwegen, 2020

*Litophyton folium* van Ofwegen, 2020

*Litophyton fusum* van Ofwegen, 2020

*Litophyton graafae* van Ofwegen, 2020

*Litophyton grandis* van Ofwegen, 2020

*Litophyton horseshoeensis* van Ofwegen, 2020

*Litophyton myrmidonensis* van Ofwegen, 2020

*Litophyton obtusum* van Ofwegen, 2020

*Litophyton oxleyensis* van Ofwegen, 2020

*Litophyton pandoraensis* van Ofwegen, 2020

*Litophyton projectum* van Ofwegen, 2020

*Litophyton pseudorowleyensis* van Ofwegen, 2020

*Litophyton rowleyensis* van Ofwegen, 2020

*Litophyton sanctuaryensis* van Ofwegen, 2020

*Litophyton simplex* van Ofwegen, 2020

*Litophyton snakeensis* van Ofwegen, 2020

*Litophyton spinulosum* van Ofwegen, 2020

*Litophyton squamatum* van Ofwegen, 2020

*Litophyton swainensis* van Ofwegen, 2020

*Litophyton territoryensis* van Ofwegen, 2020

*Litophyton verrucosum* van Ofwegen, 2020

**2021**

*Rhodolitica occulta* Breedy, van Ofwegen, McFadden &amp; Murillo-Cruz, 2021

*Unomia complanatis* Benayahu, van Ofwegen, Allais &amp; McFadden, 2021

## Appendix 2

Publications by Leen van Ofwegen (and co-authors). Sources: van Ofwegen (2007), Google Scholar Citations (https://www.researchgate.net/profile/Leen-Ofwegen/research, accessed 5 July 2021), ResearchGate (https://scholar.google.com/citations?user=DVwTdoAAAAAJ&amp;hl=en, accessed 5 July 2021.

**1982**

Krans TF, Guit FA, **van Ofwegen LP** (1982) Facies-patterns in the Lower Devonian carbonates of the Lebanza Formation (Cantabrian Mountains, Province of Palencia, NW Spain). Neues Jahrbuch für Geologie und Paläontologie Abhandlungen, Stuttgart 163(2): 192–212.

**1987**

**van Ofwegen LP** (1987) Melithaeidae (Coelenterata: Anthozoa) from the Indian Ocean and the Malay Archipelago. Zoologische Verhandelingen 239: 1–57. https://repository.naturalis.nl/pub/317750/ZV1987239001.pdf

**1989**

**van Ofwegen LP** (1989) On *Wrightella coccinea* (Ellis & Solander, 1786) and *Wrightella stiasnyi* spec. nov. (Anthozoa: Gorgonacea: Melithaeidae). Zoologische Mededelingen 63(3): 27–34. https://repository.naturalis.nl/pub/317963/ZM1989063003.pdf

**1990**

**van Ofwegen LP** (1990) Notes on the Keroeididae (Anthozoa: Gorgonacea) collected in 1963 and 1964 by the International Indian Ocean Expedition of the R/V “Anton Bruun”, in the Indian Ocean, with the description of a new species, *Lignella hartogi* spec. nov. Zoologische Mededelingen 63(13): 163–168. https://repository.naturalis.nl/pub/319109/ZM1990063013.pdf

**1991**

**van Ofwegen LP**, Vennam J (1991) Notes on Octocorallia from the Laccadives (SW India). Zoologische Mededelingen 65(9): 143–154. https://repository.naturalis.nl/pub/318729/ZM1991065009.pdf

Verseveldt J, **van Ofwegen LP** (1991) Five new species of the genus *Dendronephthya* Kükenthal, 1905 (Octocorallia: Nephtheidae) from the Indian Ocean. Zoologische Mededelingen 65(10): 155–169. https://repository.naturalis.nl/pub/318729/ZM1991065009.pdf

**1992**

**van Ofwegen LP**, Benayahu Y (1992) Notes on Alcyonacea (Octocorallia) from Tanzania. Zoologische Mededelingen 66(6): 139–154. https://repository.naturalis.nl/pub/318737/ZM1992066006.pdf

Verseveldt J, **van Ofwegen LP** (1992) New and redescribed species of *Alcyonium* Linnaeus, 1758 (Anthozoa: Alcyonacea). Zoologische Mededelingen 66(7): 155–181. https://repository.naturalis.nl/pub/318612/ZM1992066007.pdf

**1994**

**van Ofwegen LP** (1994) *Pseudothelogorgia*, a new genus of gorgonacean octocorals from the Indian Ocean. Precious Corals & Octocoral Research 3: 19–22, pls. 1–2.

**van Ofwegen LP**, Slierings M (1994) Octocorallia. In: van der Land J (Ed.) Oceanic reefs of the Seychelles (ed. J. van der Land). National Museum of Natural History, Leiden, 93–95.

**van Ofwegen LP**, Vennam J (1994) Results of the Rumphius Biohistorical Expedition to Ambon (1990). Part 3. The Alcyoniidae (Octocorallia: Alcyonacea). Zoologische Mededelingen 68(14): 135–158. https://repository.naturalis.nl/pub/319185/ZM1994068014.pdf

**1996**

**van Ofwegen LP** (1996) Octocorallia from the Bismarck Sea (part II). Zoologische Mededelingen 70(13): 207–215. https://repository.naturalis.nl/pub/318612/ZM1996070013.pdf

Vennam J, **van Ofwegen LP** (1996) Soft corals (Coelenterata: Octocorallia: Alcyonacea) from the Laccadives (SW India), with a re-examination of *Sinularia gravis* Tixier-Durivault, 1970. Zoologische Mededelingen 70(29): 437–452. https://repository.naturalis.nl/pub/318612/ZM1996070029.pdf

**1997**

Casas C, Ramil F, **van Ofwegen LP** (1997) Octocorallia (Cnidaria: Anthozoa) from the Scotia Arc, South Atlantic Ocean. I. The genus *Alcyonium* Linnaeus, 1758. Zoologische Mededelingen 71(26): 299–311. https://repository.naturalis.nl/pub/319078/ZM1997071026.pdf

den Hartog JC, **van Ofwegen LP**, van der Spoel S [Eds] (1997) Coelenterate Biology. Proceedings of the 6^th^ International Conference on Coelenterate Biology, Noordwijkerhout, The Netherlands, 16–21 July 1995. Nationaal Natuurhistorisch Museum, Leiden, 542 pp. https://repository.naturalis.nl/pub/663216/Hartog_1997.pdf

Handayani D, Edrada RA, Proksch P, Wray V, Witte L, van Ofwegen L, Kunzmann A (1997) New oxygenated sesquiterpenes from the Indonesian soft coral *Nephthea chabrolii*. Journal of Natural Products 60(7): 716–718. https://doi.org/10.1021/np960699f

**van Ofwegen LP** (1997) The present status of taxonomic knowledge of octocorals in Indonesia. In: Tomascik T, Mah AJ, Nontji A, Kasim Moosa M (Eds) The ecology of the Indonesian Seas. Part I. The Ecology of Indonesia Series VII. Periplus Editions, Hong Kong, 345–346.

**van Ofwegen LP**, Schleyer MH (1997) Corals of the South-west Indian Ocean V. *Leptophyton benayahui* gen. nov. & spec. nov. (Cnidaria, Alcyonacea) from deep reefs off Durban and on the KwaZulu-Natal south coast, South Africa. Oceanographic Research Institute Investigational Report 71: 1–12.

**1998**

Benayahu Y, **van Ofwegen LP**, Alderslade PA (1998) A case study of variation in two nominal species of *Sinularia* (Coelenterata: Octocorallia), *S. brassica* May, 1898, and *S. dura* (Pratt, 1903), with a proposal for their synonymy. Zoologische Verhandelingen 323: 277–309. https://repository.naturalis.nl/pub/317654/ZV1998323023.pdf

den Hartog JC, van Bruggen AC, Cornelius PFS, **van Ofwegen LP** [Eds] (1998) Commemorative volume for the 80^th^ birthday of Willem Vervoort in 1997. Zoologische Verhandelingen 323: 1–448.

Edrada RA, Proksch P, Wray V, Witte L, van Ofwegen L (1998) Four new bioactive diterpenes of the soft coral *Lobophytum pauciflorum* from Mindoro, Philippines. Journal of Natural Products 61(3): 358–361. https://doi.org/10.1021/np970276t

Morris LA, Christie EM, Jaspars M, **van Ofwegen LP** (1998) A bioactive secosterol with an unusual A- and B-ring oxygenation pattern isolated from an Indonesian soft coral *Lobophytum* sp. Journal of Natural Products 61(4): 538–541. https://doi.org/10.1021/np9705118

**1999**

Reinicke GB, **van Ofwegen LP** (1999) Soft corals (Alcyonacea: Octocorallia) from shallow water in the Chagos Archipelago: species assemblages and their distribution. In: Sheppard CRC, Seaward MRD (Eds) Ecology of the Chagos Archipelago. Linnean Society Occasional Publications 2. Linnean Society / Westbury Publishing, London, 67–90.

**van Ofwegen LP** (1999) *Lobophytum jasparsi* spec. nov. from Indonesia (Coelenterata: Octocorallia: Alcyonacea). Zoologische Mededelingen 73(8): 177–185. https://repository.naturalis.nl/pub/215092/ZM73_177-186.pdf

**2000**

Cinel B, Roberge M, Behrisch H, van Ofwegen L, Castro CB, Andersen RJ (2000) Antimitotic diterpenes from *Erythropodium caribaeorum* test pharmacophore models for microtubule stabilization. Organic Letters 2(3): 257–260. https://doi.org/10.1021/ol9912027

Edrada RA, Wray V, Witte L, van Ofwegen L, Proksch P (2000) Bioactive terpenes from the soft coral *Heteroxenia* sp. from Mindoro, Philippines. Zeitschrift für Naturforschung 55c: 82–86. https://doi.org/10.1515/znc-2000-1-216

**van Ofwegen LP**, Goh NKC, Chou LM (2000) The Melithaeidae: Coelenterata: Octocorallia) of Singapore. Zoologische Mededelingen 73(19): 285–304. https://repository.naturalis.nl/pub/216175/ZM73_285-304.pdf

Xu L, Patrick BO, Roberge M, Allen T, van Ofwegen L, Andersen RJ (2000) New diterpenoids from the octocoral *Pachyclavularia violacea* collected in Papua New Guinea. Tetrahedron 56: 9031–9037. https://doi.org/10.1016/S0040-4020(00)00756-0

**2001**

**van Ofwegen LP** (2001) *Sinularia vanderlandi* spec. nov. (Octocorallia: Alcyonacea) from the Seychelles. Zoologische Verhandelingen 334: 103–114. https://repository.naturalis.nl/pub/219451/ZV334_103-114.pdf

**van Ofwegen LP**, Hoeksema BW (2001) *Astrogorgia bayeri*, a new gorgonian octocoral species from South Sulawesi (Coelenterata: Octocorallia: Plexauridae). Bulletin of the Biological Society of Washington 10: 66–70.

van der Land J, van Ofwegen L, Grasshoff M (2001) Octocorallia. In: Costello MJ, Emblow C, White R (Eds.) European Register of Marine Species. A checklist of the marine species of Europe and a bibliography of guides to their identification. Patrimoines Naturels 50: 104–105.

**2002**

**van Ofwegen LP** (2002) Status of knowledge of the Indo Pacific soft coral genus *Sinularia* May, 1898 (Anthozoa: Octocorallia). Proceedings 9^th^ international Coral Reef Symposium, Bali, Indonesia 23–27 October 2000, 1: 167–171. http://coremap.or.id/downloads/IRCS9th-Ofwegen.pdf

**2003**

Hoeksema BW, **van Ofwegen LP** (2003) Indo-Malayan reef corals: a generic overview. World Biodiversity database, CD-ROM Series ETI, Amsterdam. https://indo-malayan-reef-corals.linnaeus.naturalis.nl/linnaeus_ng/app/views/introduction/topic.php?id=3292&epi=24

Ocaña O, **van Ofwegen LP** (2003) Two new genera and one new species of stoloniferous octocorals (Anthozoa: Clavulariidae). Zoologische Verhandelingen 345: 269–274. https://repository.naturalis.nl/pub/219451/ZV345_269-274.pdf

**2004**

Ocaña O, den Hartog JC, **van Ofwegen LP** (2004) Ring sea anemones, an overview (Cnidaria, Anthozoa, Actiniaria). Graellsia 60(2): 143–154. https://doi.org/10.3989/graellsia.2004.v60.i2.209

Schleyer MH, **van Ofwegen LP**, Reinicke GB (2004) Soft corals of the Western Indian Ocean and Red Sea. CD ROM. CORDIO/SIDA, Kalmar, Sweden.

**2005**

Jin P, Deng Z, Pei Y, Fu H, Li J, van Ofwegen L, Proksch P, Lin W (2005) Polyhydroxylated steroids from the soft coral *Sinularia dissecta*. Steroids 70(8): 487–493. https://doi.org/10.1016/j.steroids.2005.01.001

Li G, Deng Z, Guan H, van Ofwegen L, Proksch P, Li W (2005) Steroids from the soft coral *Dendronephthya* sp. Steroids 70(1): 13–18. https://doi.org/10.1016/j.steroids.2004.09.003

Li G, Zhang Y, Deng Z, van Ofwegen L, Proksch P, Lin W (2005) Cytotoxic cembranoid diterpenes from a soft coral *Sinularia gibberosa*. Journal of Natural Products 68(5): 649–652. https://doi.org/10.1021/np040197z

**van Ofwegen LP** (2005) A new genus of nephtheid soft corals (Octocorallia: Alcyonacea: Nephtheidae) from the Indo-Pacific. Zoologische Mededelingen 79(4): 1–236. https://repository.naturalis.nl/pub/210125/ZM79-04_001-236.pdf

**2006**

Hermanlimianto MIYT, **van Ofwegen LP** (2006) A new species of *Astrogorgia* (Coelenterata: Octocorallia: Plexauridae) from Bali. Zoologische Mededelingen 80(4): 103–108. https://repository.naturalis.nl/pub/198491/ZM80-04__103-108__Hermanlimianto.pdf

Huang X, Deng Z, Zhu X, van Ofwegen L, Proksch P, Lin W (2006) Krempenes A–D: a series of unprecedented pregnane-type steroids from the marine soft coral *Cladiella krempf*. Helvetica Chimica Acta 89: 2020–2026. https://doi.org/10.1002/hlca.200690192

Huang XP, Deng ZW, Ofwegen LV, Li J, Fu HZ, Zhu XB, Lin WH (2006) Two new pregnane glycosides from soft coral *Cladiella krempfi*. Journal of Asian Natural Products Research 8(4): 287–291. https://doi.org/10.1080/10286020500034782

McFadden CS, Alderslade P, **van Ofwegen LP**, Johnsen H, Rusmevichientong A (2006) Phylogenetic relationships within the tropical soft coral genera *Sarcophyton* and *Lobophyton* (Anthozoa, Octocorallia). Invertebrate Biology 125(4): 288–305. https://doi.org/10.1111/j.1744-7410.2006.00070.x

Stokvis FR, **van Ofwegen LP** (2006) New and redescribed encrusting species of *Alcyonium* from the Atlantic Ocean (Octocorallia: Alcyonacea: Alcyoniidae). Zoologische Mededelingen 80(4): 165–183. https://repository.naturalis.nl/pub/198498/ZM80-04__165-184__Stokvis-Ofw.pdf

**van Ofwegen LP**, Benayahu Y (2006) A new genus of paralcyoniid soft corals (Octocorallia, Alcyonacea, Paralcyoniddae) from the Indo-West Pacific. Galaxea, Journal of the Japanese Coral Reef Society 8(1): 25–37. https://doi.org/10.3755/jcrs.8.25

**van Ofwegen LP**, Cairns SD, van der Land J [Eds] (2006) Catalogue of Life: 2006. Annual Checklist – CD ROM.

**van Ofwegen LP**, Häussermann V, Försterra G (2006) A new genus of soft coral (Octocorallia: Alcyonacea: Clavulariidae) from Chile. Zootaxa 1219(1): 47–57. https://doi.org/10.11646/zootaxa.1219.1.2

Yu S, Deng Z, van Ofwegen L, Proksch P, Lin W (2006) 5,8-Epidioxysterols and related derivatives from a Chinese soft coral *Sinularia flexibilis*. Steroids 71(11–12): 955–959. https://doi.org/10.1016/j.steroids.2006.07.002

**2007**

Ma K, Li W, Fu H, Koike K, Lin W, van Ofwegen L, Fu H (2007) New 4α-methyl steroids from a Chinese soft coral *Nephthea* sp. Steroids 72(14): 901–907. https://doi.org/10.1016/j.steroids.2007.07.006

Manuputty AEW, **van Ofwegen LP** (2007) The genus *Sinularia* (Octocorallia: Alcyonacea) from Ambon and Seram (Moluccas, Indonesia). Zoologische Mededelingen 81(11): 187–216. https://repository.naturalis.nl/pub/226657/ZM81-11_187-216.pdf

**van Ofwegen LP** (2006[2007]) Annotated check list of New Caledonian soft corals. In: Payri CE, Richer de Forges B (Eds) Compendium of Marine Species from New Caledonia (2^nd^ Edn.). IRD, Nouméa New Caledonia, 139–144. https://horizon.documentation.ird.fr/exl-doc/pleins_textes/divers15-05/010059745.pdf

**van Ofwegen LP** (2007) Towards a revision of the Nephteidae (Coelenterata: Octocorallia). PhD Thesis, Leiden University, 275 pp. https://scholarlypublications.universiteitleiden.nl/handle/1887/12428

**van Ofwegen LP**, Alderslade P (2007) A new species of *Alertigorgia* (Coelenterata: Octocorallia: Anthothelidae) from the Indo Malayan region. Zoologische Mededelingen 81(13): 241–249. https://repository.naturalis.nl/pub/226659/ZM81-13_241-250.pdf

**van Ofwegen LP**, Groenenberg DSJ (2007) A centuries old problem in nephtheid taxonomy approached using DNA data (Coelenterata: Alcyonacea). Contributions to Zoology 76(3): 153–178. https://doi.org/10.1163/18759866-07603002

**van Ofwegen LP**, Häussermann V, Försterra G (2007) The genus *Alcyonium* (Octocorallia: Alcyonacea: Alcyoniidae) in Chile. Zootaxa 1607(1): 1–19. https://doi.org/10.11646/zootaxa.1607.1.1

**2008**

Cárdenas C, van Ofwegen L, Montiel A, Schories D (2008) First records of Octocorallia (Cnidaria: Anthozoa) to the Cape Horn Biosphere Reserve, Magellan Region, Chile. Anales Instituto Patagonia (Chile) 36(1): 45–52. https://doi.org/10.4067/S0718-686X2008000100004

Hoeksema BW, **van Ofwegen LP** (2008) Oceanic distribution ranges and conservation status of extant soft and hard reef coral genera. In: Leewis RJ, Janse M (Eds) Advances in Coral Husbandry in Public Aquariums. Burger’s Zoo, Arnhem. Public Aquarium Husbandry Series 2: 427–438.

Ma A, Deng Z, van Ofwegen L, Bayer M, Proksch P, Lin W (2008) Dendronpholides A-R, cembranoid diterpenes from the Chinese soft coral *Dendronephthya* sp. Journal of Natural Products 71(7): 1152–1160. https://doi.org/10.1021/np800003w

**van Ofwegen LP** (2008) The genus *Sinularia* (Octocorallia: Alcyonacea) from Bremer and West Woody Islands (Gulf of Carpentaria, Australia). Zoologische Mededelingen 82(16): 131–165. https://repository.naturalis.nl/pub/261775/ZM82-16_Ofwegen.pdf

**van Ofwegen LP** (2008) The genus *Sinularia* (Octocorallia: Alcyonacea) at Palau, Micronesia. Zoologische Mededelingen 82(51): 631–735. https://repository.naturalis.nl/pub/292118/ZM82_631-736_Ofwegen.pdf

Wen T, Ding Y, Deng Z, van Ofwegen L, Proksch P, Lin W (2008) Sinulaflexiolides A–K, cembrane-type diterpenoids from the Chinese soft coral *Sinularia flexibilis*. Journal of Natural Products 71(7): 1133–1140. https://doi.org/10.1021/np070640g

**2009**

Benayahu Y, **van Ofwegen LP** (2009) New species of *Sarcophyton* and *Lobophytum* (Octocorallia: Alcyonacea) from Hong Kong. Zoologische Mededelingen 83(30): 863–876. https://repository.naturalis.nl/pub/315883/ZM83_0863-0876_Benayahu.pdf

Häussermann V, van Ofwegen L (2009) Class Anthozoa – Anthozoans. In: Häussermann V, Försterra G (Eds.) Marine Benthic Fauna of Chilean Patagonia. Nature in Focus, Santiago, Chile, 174–176.

McFadden CS, **van Ofwegen LP**, Beckman EJ, Benayahu Y, Alderslade P (2009) Molecular systematics of the speciose Indo‐Pacific soft coral genus, *Sinularia* (Anthozoa: Octocorallia). Invertebrate Biology 128(4): 303–323. https://doi.org/10.1111/j.1744-7410.2009.00179.x

Samimi Namin K, **van Ofwegen LP** (2009) Some shallow water octocorals (Coelenterata: Anthozoa) of the Persian Gulf. Zootaxa 2058(1): 1–52. https://doi.org/10.11646/zootaxa.2058.1.1

Samimi Namin K, **van Ofwegen LP** (2009) The second observation of a live *Trimuricea* species (Octocorallia: Plexauridae). Coral Reefs 28(2): e517. https://doi.org/10.1007/s00338-009-0471-2

**van Ofwegen LP**, Breedy O, Cairns SD (2009) Octocorallia – Octocorals. In: Häussermann V, Försterra G (Eds.) Marine Benthic Fauna of Chilean Patagonia. Nature in Focus, Santiago, Chile, 177–214.

**2010**

Dautova TN, **van Ofwegen LP**, Savinkin OV (2010) New species of the genus *Sinularia* (Octocorallia: Alcyonacea) from Nha Trang Bay, South China Sea, Vietnam. Zoologische Mededelingen 84(5): 47–91. https://repository.naturalis.nl/pub/358743/ZM84_47-91_Dautova-Ofwegen-Savinkin.pdf

Ma A, Deng Z, van Ofwegen L, Bayer M, Proksch P, Lin W (2010) Additions and corrections: Dendronpholides A-R, cembranoid diterpenes from the Chinese soft coral *Dendronephthya* sp. Journal of Natural Products 73(5): e1026. https://doi.org/10.1021/np100253j

Samimi Namin K, **van Ofwegen LP** (2010) An overview of *Bebryce* (Cnidaria, Octocorallia, Plexauridae) species with tiny rosettes, with the description of a new species from the Gulf of Oman. Zoosystema 32(3): 479–493. https://doi.org/10.5252/z2010n3a9

**van Ofwegen LP**, McFadden CS (2010) A new family of octocorals (Anthozoa: Octocorallia) from Cameroon waters. Journal of Natural History 44(1): 23–29. https://doi.org/10.1080/00222930903359669

Williams DE, Amlani A, Dewi AS, Patrick BO, van Ofwegen L, Mui ALF, Andersen RJ (2010) Australin E isolated from the soft coral *Cladiella* sp. collected in Pohnpei activates the inositol 5-phosphatase SHIP1. Australian Journal of Chemistry 63(6): 895–900. https://doi.org/10.1071/CH10053

Yan P, Deng Z, van Ofwegen L, Proksch P, Lin W (2010) Lobophytones H–N, biscembranoids from the Chinese soft coral *Lobophytum pauciflorum*. Chemical and Pharmaceutical Bulletin 58(12): 1591–1595. https://doi.org/10.1248/cpb.58.1591

Yan P, Deng Z, van Ofwegen L, Proksch P, Lin W (2010) Lobophytones O–T, new biscembranoids and cembranoid from soft coral *Lobophytum pauciflorum*. Marine Drugs 8(11): 2837–2848. https://doi.org/10.3390/md8112848

Yan P, Lv Y, van Ofwegen L, Proksch P, Lin W (2010) Lobophytones A–G, New isobiscembranoids from the soft coral *Lobophytum pauciflorum*. Organic Letters 12(11): 2484–2487. https://doi.org/10.1021/ol100567d

**2011**

Benayahu Y, **van Ofwegen LP** (2011) New species of the genus *Sinularia* (Octocorallia: Alcyonacea) from Singapore with notes on the occurrence of other species of the genus. Raffles Bulletin of Zoology 59(2): 117–125. https://lkcnhm.nus.edu.sg/wp-content/uploads/sites/10/app/uploads/2017/06/59rbz117-125.pdf

Benayahu Y, **van Ofwegen LP** (2011) New species of octocorals (Coelenterata: Anthozoa) from the Penghu archipelago, Taiwan. Zoological Studies 50(3): 350–362. https://zoolstud.sinica.edu.tw/Journals/50.3/350.pdf

Hoeksema BW, van der Land J, van der Meij SET, **van Ofwegen LP**, Reijnen BT, van Soest RWM, de Voogd NJ (2011) Unforeseen importance of historical collections as baselines to determine biotic change of coral reefs: the Saba Bank case. Marine Ecology 32(2): 135–141. https://doi.org/10.1111/j.1439-0485.2011.00434.x

Lai D, Li Y, Xu M, Deng Z, van Ofwegen L, Qian P, Proksch P, Lin W (2011) Sinulariols A–S, 19-oxygenated cembranoids from the Chinese soft coral *Sinularia rigida*. Tetrahedron 67: 6018–6029. https://doi.org/10.1016/j.tet.2011.06.029

Lai D, Yu S, van Ofwegen L, Totzke F, Proksch P, Lin W (2011) 9,10-Secosteroids, protein kinase inhibitors from the Chinese gorgonian *Astrogorgia* sp. Bioorganic & Medicinal Chemistry 19(22): 6873–6880. https://doi.org/10.1016/j.bmc.2011.09.028

Reijnen BT, van der Meij SET, **van Ofwegen LP** (2011) Fish, fans and hydroids: host species of pygmy seahorses. ZooKeys 103: 1–26. https://doi.org/10.3897/zookeys.103.953

Samimi-Namin K, **van Ofwegen LP**, Wilson SC, Claereboudt MR (2011) The first in situ and shallow-water observation of the genus *Pseudothelogorgia* (Octocorallia: Keroeididae). Zoological Studies 50(2): e265. https://zoolstud.sinica.edu.tw/Journals/50.2/265.pdf

Tentori E, **van Ofwegen LP** (2011) Patterns of distribution of calcite crystals in soft corals sclerites. Journal of Morphology 272(5): 614–628. https://doi.org/10.1002/jmor.10942

**van Ofwegen LP**, Haddad MA (2011) A probably invasive new genus and new species of soft coral (Octocorallia: Alcyonacea: Clavulariidae) from Brazil. Zootaxa 3107(1): 38–46. https://doi.org/10.11646/zootaxa.3107.1.2

Yan P, Deng Z, van Ofwegen L, Proksch P, Lin W (2011) Lobophytones U–Z1, biscembranoids from the Chinese soft coral *Lobophytum pauciflorum*. Chemistry & Biodiversity 8(9): 1724–1734. https://doi.org/10.1002/cbdv.201000244

**2012**

Appeltans W, Ahyong ST, Anderson G, Angel MV, Artois T, Bailly N, Bamber R, Barber A, Bartsch I, Berta A, Blazewicz-Paszkowycz M, Bock P, Boxshall G, Boyko CB, Brandão SN, Bray RA, Bruce NL, Cairns SD, Chan T-Y, Cheng L, Collins AG, Cribb T, Curini-Galletti M, Dahdouh-Guebas F, Davie PJF, Dawson MN, De Clerck O, Decock W, De Grave S, de Voogd NJ, Domning DP, Emig CC, Erséus C, Eschmeyer W, Fauchald K, Fautin DG, Feist SW, Fransen CHJM, Furuya H, GarciaAlvarez O, Gerken S, Gibson D, Gittenberger A, Gofas S, Gómez-Daglio L, Gordon DP, Guiry MD, Hernandez F, Hoeksema BW, Hopcroft RR, Jaume D, Kirk P, Koedam N, Koenemann S, Kolb Jr B, Kristensen RM, Kroh A, Lambert G, Lazarus DB, Lemaitre R, Longshaw M, Lowry J, Macpherson E, Madin LP, Mah C, Mapstone G, McLaughlin PA, Mees J, Meland K, Messing CG, Mills CE, Molodtsova TN, Mooi R, Neuhaus B, Ng PKL, Nielsen C, Norenburg J, Opresko DM, Osawa M, Paulay G, Perrin W, Pilger JF, Poore GCB, Pugh P, Read GB, Reimer JD, Rius M, Rocha RM, Saiz-Salinas JI, Scarabino V, Schierwater B, Schmidt-Rhaesa A, Schnabel KE, Schotte M, Schuchert P, Schwabe E, Segers H, Self-Sullivan C, Shenkar N, Siegel V, Sterrer W, Stohr S, Swalla B, Tasker ML, Thuesen EV, Timm T, Todaro MA, Turon X, Tyler S, Uetz P, van der Land J, Vanhoorne B, **van Ofwegen LP**, van Soest RWM, Vanaverbeke J, Walker-Smith G, Walter TC, Warren A, Williams GC, Wilson SP, Costello MJ (2012) The magnitude of global marine species diversity. Current Biology 22(23): 2189–2202. https://doi.org/10.1016/j.cub.2012.09.036

Benayahu Y, **van Ofwegen LP** (2012) Octocorals (Cnidaria, Anthozoa) from Reunion, with a description of two new species of the genus *Sinularia* May, 1898 and notes on the occurrence of other species. Zoosystema 34(4): 673–699. https://doi.org/10.5252/z2012n4a2

Benayahu Y, **van Ofwegen LP**, Dai CF, Jeng M-S, Soong K, Shlagman A, Hsieh HJ, McFadden CS (2012) Diversity, distribution, and molecular systematics of octocorals (Coelenterata: Anthozoa) of the Penghu Archipelago, Taiwan. Zoological Studies 51(8): 1529–1548. https://zoolstud.sinica.edu.tw/Journals/51.8/1529.pdf

Breedy O, **van Ofwegen LP**, Vargas S (2012) A new family of soft corals (Anthozoa, Octocorallia, Alcyonacea) from the aphotic tropical eastern Pacific waters revealed by integrative taxonomy. Systematics and Biodiversity 10(3): 351–359. https://doi.org/10.1080/14772000.2012.707694

Chen D, Yu S, Ofwegen L, Proksch P, Lin W (2012) Anthogorgienes A–O, New guaiazulene-derived terpenoids from a Chinese Gorgonian *Anthogorgia* species, and their antifouling and antibiotic activities. Journal of Agricultural and Food Chemistry 60(1): 112–123. https://doi.org/10.1021/jf2040862

Lai D, Liu D, Deng Z, van Ofwegen L, Proksch P, Lin W (2012) Antifouling eunicellin-type diterpenoids from the gorgonian *Astrogorgia* sp. Journal of Natural Products 75(9): 1595–1602. https://doi.org/10.1021/np300404f

McFadden CS, **van Ofwegen LP** (2012) Stoloniferous octocorals (Anthozoa, Octocorallia) from South Africa, with descriptions of a new family of Alcyonacea, a new genus of Clavulariidae, and a new species of *Cornularia* (Cornulariidae). Invertebrate Systematics 26(4): 331–356. https://doi.org/10.1071/IS12035

McFadden CS, **van Ofwegen LP** (2012) A revision of the soft coral genus, *Eunephthya* Verrill, 1869 (Anthozoa: Octocorallia: Nephtheidae), with a description of four new species from South Africa. Zootaxa 3485(1): 1–25. https://doi.org/10.11646/zootaxa.3485.1.1

Samimi-Namin K, **van Ofwegen LP** (2012) The octocoral fauna of the Gulf. In: Riegl BM, Purkis SJ (Eds) Coral Reefs of the Gulf: Adaptation to Climatic Extremes. Springer, Dordrecht, 225–252. https://doi.org/10.1007/978-94-007-3008-3

Shen S, Zhu H, Chen D, Liu D, van Ofwegen L, Proksch P, Lin W (2012) Pavidolides A–E, new cembranoids from the soft coral *Sinularia pavida*. Tetrahedron Letters 53(43): 5759–5762. https://doi.org/10.1016/j.tetlet.2012.08.049

Shi H, Yu S, Liu D, van Ofwegen L, Proksch P, Lin W (2012) Sinularones A–I, new cyclopentenone and butenolide derivatives from a marine soft coral *Sinularia* sp. and their antifouling activity. Marine Drugs 10(6): 1331–1334. https://doi.org/10.3390/md10061331

**van Ofwegen LP**, Benayahu Y (2012) Two new species and a new record of the genus *Sinularia* (Octocorallia, Alcyonacea) from the Penghu archipelago, Taiwan. Zoological Studies 51(3): 283–398. https://zoolstud.sinica.edu.tw/Journals/51.3/383.pdf

Williams GC, Hoeksema BW, **van Ofwegen LP** (2012) A fifth morphological polyp in pennatulacean octocorals, with a review of polyp polymorphism in the genera *Pennatula* and *Pteroeides* (Anthozoa: Pennatulidae). Zoological Studies 51(7): 1006–1017. https://zoolstud.sinica.edu.tw/Journals/51.7/1006.pdf

**2013**

Chen D, Chen W, Liu D, van Ofwegen L, Proksch P, Lin W (2013) Asteriscane-type sesquiterpenoids from the soft coral *Sinularia capillosa*. Journal of Natural Products 76(9): 1753–1763. https://doi.org/10.1021/np400480p

Lai D, Geng Z, Deng Z, van Ofwegen L, Proksch P, Lin W (2013) Cembranoids from the soft coral Sinularia rigida with antifouling activities. Journal of Agricultural and Food Chemistry 61(19): 4585–4592. https://doi.org/10.1021/jf401303q

McFadden CS, **van Ofwegen LP** (2013) A second, cryptic species of the soft coral genus Incrustatus (Anthozoa: Octocorallia: Clavulariidae) from Tierra del Fuego, Argentina, revealed by DNA barcoding. Helgoland Marine Research 67(1): 137–147. https://doi.org/10.1007/s10152-012-0310-7

McFadden CS, **van Ofwegen LP** (2013) Molecular phylogenetic evidence supports a new family of octocorals and a new genus of Alcyoniidae (Octocorallia, Alcyonacea). ZooKeys 346: 59–83. https://doi.org/10.3897/zookeys.346.6270

Santodomingo N, Reyes J, Flórez P, Chacón-Gómez IC, **van Ofwegen LP**, Hoeksema BW (2013) Diversity and distribution of azooxanthellate corals in the Colombian Caribbean. Marine Biodiversity 43(1): 7–22. https://doi.org/10.1007/s12526-012-0131-6

**van Ofwegen LP**, Benayahu Y, McFadden CS (2013) *Sinularia leptoclados* (Ehrenberg, 1834) (Cnidaria: Octocorallia) re-examined. ZooKeys 272: 29–59. https://doi.org/10.3897/zookeys.272.4406

Xi Z, Bie W, Chen W, Liu D, van Ofwegen L, Proksch P, Lin W (2013) Sarcophyolides B–E, new cembranoids from the soft coral *Sarcophyton elegans*. Marine Drugs 11(9): 3186–3196. https://doi.org/10.3390/md11093186

Xi Z, Bie W, Chen W, Liu D, van Ofwegen L, Proksch P, Lin W (2013) Sarcophytolides G–L, new biscembranoids from the soft coral *Sarcophyton elegans*. Helvetica Chimica Acta 96(12): 2218–2227. https://doi.org/10.1002/hlca.201300086

**2014**

Chen D, Cheng W, Liu D, van Ofwegen L, Proksch P, Lin W (2014) Capillosananes S–Z, new sesquiterpenoids from the soft coral *Sinularia capillosa*. Tetrahedron Letters 55(19): 3077–3082. https://doi.org/10.1016/j.tetlet.2014.03.132

Liu Z, Cheng W, Liu D, van Ofwegen L, Proksch P, Lin W (2014) Capnosane-type cembranoids from the soft coral *Sarcophyton trocheliophorum* with antibacterial effects. Tetrahedron 70(45): 8703–8713. https://doi.org/10.1016/j.tet.2014.09.034

McFadden CS, Brown AS, Brayton C, Hunt CB, **van Ofwegen LP** (2014) Application of DNA barcoding to biodiversity studies of shallow-water octocorals: molecular proxies agree with morphological estimates of species richness in Palau. Coral Reefs 33(2): 275–286. https://doi.org/10.1007/s00338-013-1123-0

Reijnen BT, Hoeksema BW, **van Ofwegen LP**, van der Meij SET (2014) Soft corals and sea fans: Symbioses with Octocorallia studied. In: Naturalis Research and Education Report 2009–2012, Naturalis Biodiversity Center. Leiden, 96–97.

Reijnen BT, McFadden CS, Hermanlimianto YT, **van Ofwegen LP** (2014) A molecular and morphological exploration of the generic boundaries in the family Melithaeidae (Coelenterata: Octocorallia) and its taxonomic consequences. Molecular Phylogenetics and Evolution 70: 383–401. https://doi.org/10.1016/j.ympev.2013.09.028

van der Meij SET, Hoeksema BW, **van Ofwegen LP**, Reijnen BT, van Soest RWM, de Voogd NJ (2014) Changing marine faunas: Importance of historical collections. Naturalis Research and Education Report 2009–2012, Naturalis Biodiversity Center. Leiden, 94–95.

**van Ofwegen LP**, Hermanlimianto MIYT (2014) Siboga plexaurids (Coelenterata: Octocorallia) re-examined. Zoologische Mededelingen 88(3): 19–58. https://repository.naturalis.nl/pub/516157/ZM88-03_19-58_Ofwegen.pdf

**van Ofwegen LP**, Aurelle D, Sartoretto S (2014) A new genus of soft coral (Cnidaria, Octocorallia) from the Republic of Congo (Pointe-Noire Region). ZooKeys 462: 1–10. https://doi.org/10.3897/zookeys.462.8533

**2015**

Fransen CHJM, ten Hove HA, Lau YW, van der Meij SET, **van Ofwegen LP**, Rauch C, Reijnen BT, van Tienderen KM, Hoeksema BW (2015) New biodiversity research at Curaçao. In: Naturalis Research and Education Report 2013–2014, Naturalis Biodiversity Center, Leiden, 94–95.

Huang Q, Cheng W, Long H, Liu H, van Ofwegen L, Lin W (2015) Subergane-type sesquiterpenes from gorgonian coral *Subergorgia suberosa* with antibacterial activities. Helvetica Chimica Acta 98(9): 1202–1209. https://doi.org/10.1002/hlca.201500086

Matsumoto AK, **van Ofwegen LP** (2015) Melithaeidae of Japan (Octocorallia, Alcyonacea) re-examined with descriptions of 11 new species. ZooKeys 522: 1–127. https://doi.org/10.3897/zookeys.522.10294

Mizrahi GA, Shemesh E, van Ofwegen L, Tchernov D (2015) First record of *Aequorea macrodactyla* (Cnidaria, Hydrozoa) from the Israeli coast of the eastern Mediterranean Sea, an alien species indicating invasive pathways. NeoBiota 26: 55–70. https://doi.org/10.3897/neobiota.26.8278

Zhang NX, Tang XL, van Ofwegen L, Xue L, Song WJ, Li PL, Li GQ (2015) Cyclopentenone derivatives and polyhydroxylated steroids from the soft coral *Sinularia acuta*. Chemistry and Biodiversity 12(2): 273–283. https://doi.org/10.1002/cbdv.201400044

**2016**

Bayer FM, **van Ofwegen LP** (2016) The type specimens of *Bebryce* (Cnidaria, Octocorallia, Plexauridae) re-examined, with emphasis on the sclerites. Zootaxa 4083(3): 301–358. https://doi.org/10.11646/zootaxa.4083.3.1

Cheng W, Ren J, Huang Q, Long H, Jin H, Zhang L, Liu H, van Ofwegen L, Lin W (2016) Pregnane steroids from a gorgonian coral *Subergorgia suberosa* with anti-flu virus effects. Steroids 108: 99–104. https://doi.org/10.1016/j.steroids.2016.02.003

Matsumoto AK, **van Ofwegen LP** (2016) The genus *Bebryce* (Cnidaria, Octocorallia, Plexauridae) at Japan, with descriptions of three new species. ZooKeys 587: 1–20. https://doi.org/10.3897/zookeys.587.8188

Matsumoto AK, **van Ofwegen LP** (2016) Species of *Elasmogorgia* and *Euplexaura* (Cnidaria, Octocorallia) from Japan with a discussion about the genus *Filigella*. ZooKeys 589: 1–21. https://doi.org/10.3897/zookeys.589.8361

Samimi-Namin K, **van Ofwegen LP** (2016) Overview of the genus *Briareum* (Cnidaria, Oc­tocorallia, Briareidae) in the Indo-Pacific, with the description of a new species. ZooKeys 557: 1–44. https://doi.org/10.3897/zookeys.557.6298

Samimi-Namin K, **van Ofwegen LP** (2016) A revision of *Trimuricea* Gordon, 1926 (Cnidaria: Octocorallia: Plexauridae) with the description of six new species. Zootaxa 4105(1): 1–44. https://doi.org/10.11646/zootaxa.4105.1.1

Samimi-Namin K, **van Ofwegen LP**, McFadden CS (2016) A new species of *Melithaea* (Anthozoa, Octocorallia, Melithaeidae) from the Oman Sea, off Oman. ZooKeys 623: 15–29. https://doi.org/10.3897/zookeys.623.10045

Sampaio Í, Stokvis FR, **van Ofwegen LP** (2016) New name for the soft coral *Alcyonium rubrum* Stokvis & van Ofwegen, 2006 (Alcyonacea, Alcyoniidae): *Alcyonium burmedju* nom. n. ZooKeys 619: 163–165. https://doi.org/10.3897/zookeys.619.10086

**van Ofwegen LP** (2016) The genus *Litophyton* Forskål, 1775 (Octocorallia, Alcyonacea, Nephtheidae) in the Red Sea and the western Indian Ocean. ZooKeys 567: 1–128. https://doi.org/10.3897/zookeys.567.7212

**van Ofwegen LP**, McFadden CS, Benayahu Y (2016) *Sinularia polydactyla* (Ehrenberg, 1834) (Cnidaria, Octocorallia) re-examined, with the description of a new species. ZooKeys 581: 71–126. https://doi.org/10.3897/zookeys.581.7455

**2017**

Benayahu Y, McFadden CS, Shoham E, **van Ofwegen LP** (2017) Search for mesophotic octocorals (Cnidaria, Anthozoa) and their phylogeny. II. A new zooxanthellate species from Eilat, northern Red Sea. ZooKeys 676: 1–12. https://doi.org/10.3897/zookeys.676.12751

Cheng W, Ji M, Li X, Ren J, Yin F, van Ofwegen L, Yu S, Chen X, Lin W (2017) Fragilolides A–Q, norditerpenoid and briarane diterpenoids from the gorgonian coral *Junceella fragilis*. Tetrahedron 73(17): 2518–2528. https://doi.org/10.1016/j.tet.2017.03.037

Cheng W, Li X, Yin F, van Ofwegen L, Lin W (2017) Halogenated briarane diterpenes with acetyl migration from the gorgonian coral *Junceella fragilis*. Chemistry & Biodiversity 14(5): e1700053. https://doi.org/10.1002/cbdv.201700053

Cheng W, Liu Z, Yu Y, van Ofwegen L, Proksch P, Yu S, Lin W (2017) An unusual spinaceamine-bearing pregnane from a soft coral *Scleronephthya* sp. inhibits the migration of tumor cells. Bioorganic & Medicinal Chemistry Letters 27(12): 2736–2741. https://doi.org/10.1016/j.bmcl.2017.04.058

McFadden CS, **van Ofwegen LP** (2017) Revisionary systematics of the endemic soft coral fauna (Octocorallia: Alcyonacea: Alcyoniina) of the Agulhas Bioregion, South Africa. *Zootaxa* 4363(4): 451–488. https://doi.org/10.11646/zootaxa.4363.4.1

**2018**

Benayahu Y, **van Ofwegen LP**, McFadden CS (2018) Evaluating the genus *Cespitularia* Milne Edwards & Haime, 1850 with descriptions of new genera of the family Xeniidae (Octocorallia, Alcyonacea). ZooKeys 754: 63–101. https://doi.org/10.3897/zookeys.754.23368

Benayahu Y, **van Ofwegen LP**, Dai CF, Jeng MS, Soong K, Shlagman A, Du SW, Hong P, Imam NH, Chung A, Wu T, McFadden CS (2018) The octocorals of Dongsha Atoll (South China Sea): an iterative approach to species identification using classical taxonomy and molecular barcodes. Zoological Studies 57: 1–50. https://doi.org/10.6620/ZS.2018.57-50

Chu MJ, Tang XL, Han X, Li T, Luo XC, Jiang MM, van Ofwegen L, Luo LZ, Zhang G, Li PL, Li GQ (2018) Metabolites from the Paracel Islands soft coral Sinularia cf. molesta. Marine Drugs 16(12): e517. https://doi.org/10.3390/md16120517

Cordeiro RTS, **van Ofwegen LP** (2018) A new name for *Dendronephthya kukenthali* Gravier, 1908 (Octocorallia, Nephtheidae): *Dendronephthya perezi* nom. n. Zootaxa 4508(4): 576–578. https://doi.org/10.11646/zootaxa.4508.4.6

Lau YW, Stokvis FR, **van Ofwegen LP**, Reimer JD (2018) Stolonifera from shallow waters in the north-western Pacific: a description of one new genus and two new species within the Arulidae (Anthozoa, Octocorallia). ZooKeys 790: 1–19. https://doi.org/10.3897/zookeys.790.28875

Qin GF, Tang XL, Sun YT, Luo XC, Zhang J, van Ofwegen L, Sung PJ, Li PL, Li, GQ (2018) Terpenoids from the soft coral *Sinularia* sp. collected in Yongxing Island. Marine Drugs 16(3): e127. https://doi.org/10.3390/md16040127

Tuti Y, **van Ofwegen LP** (2018) Gorgonians in Indonesian Waters. Media Sains National, Bogor, Indonesia, 89 pp. http://oseanografi.lipi.go.id/hasilpenelitian/lihatpdf/43

**2019**

Matsumoto AK, **van Ofwegen LP**, Bayer FM (2019) A revision of the genus *Calcigorgia* (Cnidaria, Octocorallia, Acanthogorgiidae) with the description of three new species. Zootaxa 4571(1): 1–27. https://doi.org/10.11646/zootaxa.4571.1.1

Zhang J, Tang X, Han X, Feng D, Luo X, **van Ofwegen LP**, Li P, Li G (2019) Sarcoglaucins A–I, new antifouling cembrane-type diterpenes from the South China Sea soft coral *Sarcophyton glaucum*. Organic Chemistry Frontiers 6(12): 2004–2013. https://doi.org/10.1039/C9QO00386J

**2020**

Lau YW, Stokvis FR, van Ofwegen L, Reimer JD (2020) Corrigenda: *Hanah*, a replacement name for *Hana* Lau, Stokvis, Ofwegen & Reimer, 2018 (preoccupied name). ZooKeys 918: 161–162. https://doi.org/10.3897/zookeys.918.51123

**van Ofwegen LP** (2020) The genus *Litophyton* Forskål, 1775 (Octocorallia, Alcyonacea, Nephtheidae) from Australia. Zootaxa 4764(1): 1–131. https://doi.org/10.11646/zootaxa.4764.1.1

**2021**

Benayahu Y, **van Ofwegen LP**, Ruiz Allais JP, McFadden CS (2021) Revisiting the type of *Cespitularia stolonifera* Gohar, 1938 leads to the description of a new genus and a species of the family Xeniidae (Octocorallia, Alcyonacea). Zootaxa 4964(2): 330–344. https://doi.org/10.11646/zootaxa.4964.2.5

Breedy O, **van Ofwegen LP**, McFadden CS, Murillo-Cruz C (2021) *Rhodolitica* on rhodoliths: a new stoloniferan genus (Anthozoa, Octocorallia, Alcyonacea). ZooKeys 1032: 63–77. https://doi.org/10.3897/zookeys.1032.63431

Wang Z, Li PL, Luo XC, Wang Q, van Ofwegen L, Tang XL, Li GQ (2021) Terpenoids from the South China Sea soft coral *Sinularia multiflora*. Natural Product Research 35(14): 2395–2402. https://doi.org/10.1080/14786419.2019.1678615

**Online but not published in final version**

Liu M, Li P, Luo X, van Ofwegen L, Tang X, Li G (2020) Sesquiterpenoids from the soft coral *Lemnalia* sp. Natural Product Research. https://doi.org/10.1080/14786419.2020.1736062

**Online source**

Cordeiro R, McFadden C, van Ofwegen L, Williams G (2021) World List of Octocorallia. Accessed through http://www.marinespecies.org/
